# Mountain and Malarious Fevers Produced by the Same Cause

**Published:** 1851-07

**Authors:** Ira E. Oatman

**Affiliations:** Dundee, Ill’s.


### ARTICLE V.

### *Mountain and Malarious Fevers produced by the same Cause:*

By Ira E. Oatman, M.D., of Dundee, Ill's.

In passing over one thousand miles of the vast plains towards the Pacific, the earlier emigrants of 1849, enjoyed great freedom from fevers. But while going through the South Pass of the Rocky Mountains, some of them experienced extreme dyspnœa, which was increased by exercise. The altitude was 8,500 feet above the level of the sea. This dyspnœa so resembled that I had observed in congestive fevers in Illinois, where the lungs were chiefly affected, that I was induced to enquire if they were produced by the same cause. We began to descend the same day, and were in lower altitude; but still several thousand feet above the level of the sea. Within a few days, (I lost my Journal,) one-fourth of our company were taken with what is called the "Mountain Fever."

They were attacked with depression of spirits, languor, and debility, with extreme aching in the fore-head and lumbar regions of the back, thirst, coldness and numbness of the extremeties, and slight chills. These were followed by an increase of thirst and pain, with febrile reaction—generally high, and in some cases delirium. If not energetically treated, the remission was very light in the morning. The cases yielded in from one to three days, to six or eight grains of Sulph. Quinine and one-fourth to one-third of a grain of Sulph. Morphine three or four times a day, after a mercurial crthartic. The convalescence, was as rapid as is usual in this country, after such fevers.

Fat bacon and coffee had been the staple articles of food, since we left home; and such was our mode of life, that we partook of them in no sparing quantities. These being highly carbonacious articles, and the weather rather warm, there was evidently an accumulation of carbon in the system. While the same quantities were still ingested, we ascended to an altitude, (8,500 feet,) where the atmosphere was so rare, that, with deep and frequent inspirations, there was not sufficient oxygen taken into the circulation, to maintain the vital actions during active exercise. Hence the dyspnœa and sense of impending suffocation. The carbon, not meeting the proper eliments with which to combine, could not be eliminated from the system through the natural avenues. Consequently, a vast accumulation would take place in a short time. The coldness of the nights in this altitude, checking the insensible prespiration, would produce a pathological state of the blood, which would derange, consecutively, the functions of the liver, kidneys, etc., through which the carbon is eliminated. The blood, now loaded with carbon, and imperfectly hæmatized; failing thereby to stimulate the cerebral and nervous functions, that state of depression and languor would follow, which results in chills and all the phenomena of fever.

Here the country was dry and the air salubrious; and to the closest observer, there was no local cause of disease. Yet here only did we suffer from fevers; and here only did we see the graves of former emigrants. It seemed reasonable to attribute these fevers to an excess of carbon in the system; there being no other appreciable cause.

Let us now examine if fevers called malarious are produced by the same cause. Epidemics of these fevers, occur after protracted heat and drought in marshy and alluvial localities. And according to Dr. Ferguson and others, in the dried up beds of streams; and where absorbing surfaces had been flooded and had become dry. The invariable conditions of protracted heat and drought, would so rarify the air, that the carbon not meeting oxygen in adequate proportions for combination, would accumulate in the system; and if the diet used was carbonacious, (and soldiers generally use such,) the quantities would be increased. If these conditions obtained where there was vegetable matter in an advanced stage of decomposition, as is usually the case where these diseases prevail, then, through the lungs, would large additions be made to the already excessive quantity of carbon in the system. In the "dried up beds of streams," and where "absorbing surfaces" had been flooded, and had become dry, we would expect to find the remains of decaying vegetable matter largely in the former case, and to some extent in the latter, although not perceptible perhaps, to the eye. But if not as alleged, there were the invariable conditions of protracted heat and drought, or a high altitude, and probably carbonacious diet, which would produce the same pathological condition of the fluids. The California emigrants above referred to, had neither drought or considerable heat; nor yet were they exposed to alluvial lands.

made bare, or grounds annually flooded and now dry and absorbing, with the "vegetation utterly burned up." And yet they had all the phenomena of endemic malarious fever, which was answerable to the same treatment.

This being the true ætiology of these diseases, we may better understand the modus operandi of venesection, practiced for the cure of intermittents in the cold stage,—how Quinine, and Morphia, and even active stimulants, remove all the phenomena of fever, by equalizing the circulation, quieting nervous irritability, promoting prespiration, and the secretions of the liver, kidneys, etc. And how the recuperative powers of the system are exerted to free the blood from the surplus carbon, by increasing the fullness and frequency of the respirations, by the vigorous circulation, etc., in fever; making powerful (though sometime abortive) efforts to excite all the organs to vigorous action, through which the carbon is eliminated from the body. Or if there is a relative deficiency of nitrogen in the cerebrum and nervous system, how that is supplied by quinia, and other nitrogenized substances.

Having seen no very satisfactory theory of the chemical nature of this indefinable agent we call malaria, I submit the results of my observations, hoping that they may, at least, throw some light upon the further elucidation of this subject, which has so long been a desideratum in medicine.

There is a highly interesting article in the first volume of the North-Western Med. and Surg. Journal, I find, in reference to this theory of the ætiology of malarious diseases, by S. G. Armor, M.D., then of Rockford, Ill.

Other theories are advocated with plausibility, by eminent men of the west. The progress of medical science is such, that we may hope to be able to demonstrate the true chemical cause of our malarious diseases, at no very remote period.

*April* 14, 1851.

